# The *Trichoderma harzianum* Kelch Protein ThKEL1 Plays a Key Role in Root Colonization and the Induction of Systemic Defense in Brassicaceae Plants

**DOI:** 10.3389/fpls.2019.01478

**Published:** 2019-11-15

**Authors:** Jorge Poveda, Rosa Hermosa, Enrique Monte, Carlos Nicolás

**Affiliations:** ^1^Department of Botany and Plant Physiology, Spanish-Portuguese Institute for Agricultural Research (CIALE), University of Salamanca, Salamanca, Spain; ^2^Department of Microbiology and Genetics, Spanish-Portuguese Institute for Agricultural Research (CIALE), University of Salamanca, Salamanca, Spain

**Keywords:** Brassicaceae, rapeseed, *Arabidopsis*, tomato, myrosinase, root colonization, *Thkel1*, *Trichoderma*

## Abstract

The fungal genus *Trichoderma* includes strains with biocontrol and/or biostimulant potential and is recognized as a source of genes with biotechnological value. In a previous study the Kelch domain protein, encoded by the *Thkel1* gene of *Trichoderma harzianum* T34, was found to confer tolerance to salt stress when expressed in plants of *Arabidopsis thaliana*. In the present work, we have overexpressed *Thkel1* in rapeseed plants in order to generate an additional biotechnological tool for analyzing the role of this gene in *Trichoderma*-plant interactions. The overexpression of this gene in Brassicaceae plants improves responses to pathogens through the induction of systemic defenses mediated by jasmonic acid, facilitates root colonization by modulating the myrosinase activity, and, as a result, increases plant productivity. These effects were also observed in *Thkel1* overexpressing plants subjected to abiotic stress conditions. Additionally, the differences detected in root colonization levels by *T. harzianum* wild type and *Thkel1* silenced transformants between *Arabidopsis* or rapeseed and tomato plants indicate that ThKEL1 interacts in different ways in Brassicaceae and non-Brassicaceae plants.

## Introduction

*Trichoderma* is a genus of soil-borne filamentous fungi able to colonize diverse substrates under different environmental conditions that includes species widely used as biocontrol agents in agriculture. In particular their capacity to antagonize plant pathogens through different mechanisms such as mycoparasitism, antibiosis, and competition is worth noting (Lorito et al., 2010), as well as the ability of some strains to stimulate plant growth and development and induce plant defense against biotic and abiotic stresses (Hermosa et al., 2012; Hermosa et al., 2013; Rubio et al., 2014; Rubio et al., 2017). These effects are the consequence of different metabolic changes modulated by the intimate interaction between *Trichoderma* and roots. The molecular dialog between this beneficial fungus and plants has been studied intensively through the use of techniques such as proteomics (Marra et al., 2006), metabolomics (Vinale et al., 2008), transcriptomics and co-expression analysis (Morán-Diez et al., 2012; Brotman et al., 2013), secretomics (Mendoza-Mendoza et al., 2018), and confocal microscopy (Carrero-Carrón et al., 2018). Considering the different mechanisms of biocontrol that have been identified, it is clear that this process involves many genes and gene products which can be isolated and cloned to produce disease-resistant genetically modified (GM) crops. A pioneer work showed that a *Trichoderma* chitinase gene could be functionally expressed in tobacco and potato plants conferring beneficial characteristics, mainly in disease control (Lorito et al. 1998). Subsequently, many examples of transformed plants with other *Trichoderma* genes, such as those encoding a cellobiohydrolase or a glutathione transferase in tobacco (Dai et al., 1999; Dixit et al., 2011), α and β glucanases in *Arabidopsis* or pearl millet (Calo et al., 2006; O`Kennedy et al., 2011), a xylanase in fescue (Buanafina et al., 2012), or a heat shock protein 70 in *Arabidopsis* (Montero-Barrientos et al., 2010) have been reported.

In a previous study, we isolated and characterized the *Thkel1* gene from *T. harzianum* (Hermosa et al., 2011) and observed that this gene: i) codes for a protein which showed similarity to plant nitrile-specifier proteins (NSPs) and epithiospecifier proteins (ESPs), which when interacting with myrosinases convert glucosinolates to both simple nitriles and epithionitriles depending on the glucosinolate structure (Kissen and Bones, 2009); and ii) was related to the β-glucosidase activity of *T. harzianum. Thkel1* codes for the ThKEL1 protein which comprises five Kelch-repeat domains (Hermosa et al., 2011). Kelch-like family proteins are highly conserved in evolution and are considered to play important roles in cell morphology (Dhanoa et al., 2013; Zhang et al., 2013), as well as in protein-protein interactions. They act as substrate adaptor proteins for the SCF (Skp1, Cullin, F-box containing complex) ubiquitin ligase complex, catalyzing the ubiquitination of proteins destined for degradation in the 26S proteasome. In plants, this class of enzymes play a key role in many physiological processes, including the circadian clock, plant growth and development, defense responses, metabolism of cell wall lignification, activation of phytohormones, and secondary metabolism or fruit ripening (Hassan et al., 2015). We also observed that *Thkel1* improves *T. harzianum* adaptation to abiotic stress. Furthermore, the transformation of *Arabidopsis* plants with this gene produced an enhanced response to salt and osmotic stresses through the modulation of β-glucosidase activity (Hermosa et al., 2011).

The aim of this work is to build upon what is already known about the role of the *T. harzinum Thkel1* gene in *Trichoderma*-plant root colonization. For this purpose, we analyzed the interaction of *T. harzianum* with *Arabidopsis*, rapeseed, and tomato plants using previously generated modified organisms (Hermosa et al., 2011), such as the *Thkel1*-silenced transformants of *T. harzianum* and transgenic plants of *Arabidopsis*, expressing this gene, as well as *Thkel1* rapeseed transgenic plants obtained in the present work. Thus, we have analyzed plant responses to foliar pathogens, such as *B. cinerea* or *P. lingam*, as well as the expression levels of several defense-marker genes. Finally, we determined the degree of root colonization using *Trichoderma* wild type and *Thkel1*-silenced mutant strains, myrosinase activity, and rapeseed productivity, both under abiotic stress and control conditions.

## Material and Methods

### Plant Material

The *Arabidopsis thaliana* ecotype Col-0 and its previously described (Hermosa et al., 2011) M2 transgenic line expressing the *Thkel1* gene from *T. harzianum* T34, named throughout the article as AtKel2, have been used. Since the three independent *Thkel1* transgenic lines obtained in our laboratory exhibited similar phenotypes, only results from AtKel2 are shown along the manuscript. *Brassica napus* cv. Jura and *Solanum lycopersicum* cv. Marmande were the other plants used in this study. Seeds were surface sterilized as previously described (Rubio et al., 2017).

### Rapeseed Transformation

To generate the vector construct used for rapeseed transformation, we used the GATEWAY^™^ methodology (Karimi et al., 2002). Two primers were designed to introduce the attB1 and attB2 recombination sites at the 5´ and 3´ ends, respectively of the pDONR201 plasmid (**Table 1**), obtaining the pENTR201 plasmid. Finally, this plasmid was used to generate the pKGWFS7-*Thkel1* construct, the DESTINY plasmid, that contains the T-DNA region that was transferred to *Agrobacterium tumefaciens* C58C1 by electroporation (Mersereau et al., 1990). Rapeseed plants were transformed by the floral dip method and transgenic seedlings were selected on kanamycin medium (50 µg/ml).

**Table 1:** Oligonucleotides used in this work.

Code	Sequence (5’-3’)	Use
Act-T-F	ATGGTATGGGTCAGAAGGA	Endogenous *Trichoderma* gene
Act-T-R	ATGTCAACACGAGCAATGG	
35S-GTW-F	CTTCGCAAGACCCTTCCTCT	Testing rapeseed transformation with *Thkel1* gene
Thkel1-R	GGGGACCACTTTGTACAAGA​AAGCTGGGTCTTACAAAAAG​TCCAACCTCC	
ThkelQ-F	ACGGCACAAGCTCCACTTG	*Thkel1* gene expression
ThkelQ-R	TGCGGGACGAGGGATAGAC	
Act-Bn-F	CCCTGGAATTGCTGACCGTA	Endogenous rapeseed gene
Act-Bn-R	TGGAAAGTGCTGAGGGATGC	
Act-At-F	CTCCCGCTATGTATGTCGCC	Endogenous *Arabidopsis* gene
Act-At-R	TTGGCACAGTGTGAGACACAC	
ICS1-At-F	GATCTAGCTAACGAGAACGG	Synthesis gene of SA in *Arabidopsis*
ICS1-At-R	CATTAAACTCAACCTGAGGGAC	
PR-1-At-F	GGCTAACTACAACTACGCTG	Response gene to SA in *Arabidopsis*
PR-1-At-R	GGCTTCTCGTTCACATAATTC	
LOX1-At-F	GTAAGCTCTGATGTTACTGATTC	Synthesis gene of JA in *Arabidopsis*
LOX1-At-R	CTGCGGTTAACGACGTGATTG	
VSP2-At-F	GTTAGGGACCGGAGCATCAA	Response gene to JA in *Arabidopsis*
VSP2-At-R	TCAATCCCGAGCTCTATGATGTT	
Act-Sl-R	CACCACTGCTGAACGGGAA	Endogenous tomato gene
Act-Sl-R	GGAGCTGCTCCTGGCAGTTT	
ICS1-Sl-F	GTTCCTCTCCAAGAATGTCC	Synthesis gene of SA in tomato
ICS1-Sl-R	TCCTTCAAGCTCATCAAACT	
PR-1-Sl-F	CCTCAAGATTATCTTAACGCTC	Response gene to SA in tomato
PR-1-Sl-R	TACCATTGCTTCTCATCAACC	
LOX1-Sl-F	GCCTCTCTTCTTGATGGAG	Synthesis gene of JA in tomato
LOX1-Sl-R	GTAGTGAGCCACTTCTCCAA	
EIN2-Sl-F	GTTGCTAAGTGATGCTGTA	Response gene to JA/ET in tomato
EIN2-Sl-R	CGCTCAAGCATGCTGGGCC	

T1 kanamycin-resistant seeds were recovered and Polymerase Chain Reaction (PCR) analyzed using the specific oligonucleotides 35S-GTW-F and Thkel1-R (**Table 1**). Selected plants were continued until T3 and were considered homozygous. Two independent transgenic lines, namely BnKel1 and BnKel2, were selected for the subsequent analysis.

As expected, the transgene was detected in the transformed plants and the expression levels of *Thkel1* gene in both *Arabidopsis* and rapeseed transgenic plants are shown in **Figure S1**.

### Plant Growth and Conditions

Seeds were grown on Murashige and Skoog (MS) (Duchefa, Haarlem, Netherlands) solid medium (agar 1%) with sucrose (1%) in a growth chamber at 22°C, 40% relative humidity (RH), and a 16 h light/8 h dark photoperiod at 80–100 µE m^−2^ s^−1^, for 7 (rapeseed), 10 (*Arabidopsis*), and 16 (tomato) days. *Arabidopsis* seedlings were individually transferred to 0.2 L-pots and rapeseed and tomato seedlings to 5 L-pots, containing a mixture of peat/vermiculite (3:1) and maintained in a greenhouse at 22 ± 2°C as previously described (Montero-Barrientos et al., 2010). Hydroponic culture of *A. thaliana* was also carried out as previously described (Alonso-Ramírez et al., 2014).

### *Trichoderma* Cultures and Inoculation

*T. harzianum* CECT 2413 (Spanish Type Culture Collection, Valencia, Spain, referred to as strain T34) was used throughout this study. Moreover, the K4 and K10 *T. harzianum* transformants, obtained by silencing with self-complementary “hairpin” RNAs (intron hairpin RNA [ihpRNA]) of the *Thkel1* gene, together with the transformation control strain ThJL43 were used (Hermosa et al., 2011). Strains were routinely grown on potato-dextrose-agar (PDA, Sigma-Aldrich, Madrid, Spain) in the dark at 28°C and the spores were stored at −80°C in a 20% glycerol solution. Spores were harvested from 7-day-old PDA dishes as previously described (Rubio et al., 2014).

*Arabidopsis* was inoculated with *T. harzianum* strains in the hydroponic culture following the method described by Alonso-Ramírez et al. (2014). Rapeseed and tomato treated with *T. harzianum* T34 and its transformants were root inoculated with 1 ml of a conidial suspension containing 2 × 10^7^ spore ml^−1^, determined using a hemocytometer, 1 week after the seedlings were transplanted.

### Quantification of Root Colonization

The quantification of T34 DNA in the roots of rapeseed, *Arabidopsis* and tomato plants was performed by quantitative PCR (qPCR) as previously described (Morán-Diez et al., 2009; Alonso-Ramírez et al., 2014), with some modifications. Roots were collected during the formation of the floral primordia in 10- (rapeseed) and 7- (tomato) week-old plants, and 7 days after inoculation with the fungus in the case of *Arabidopsis* hydroponic culture. DNA was extracted using the cetyl-trimethyl-ammonium bromide (CTAB) extraction method (Dellaporta et al., 1983). A mix was prepared in a 10 µl volume using 5 µl of Brilliant SYBR Green QPCR Master Mix (Roche), 10 ng of DNA, the forward and reverse primers at a final concentration of 100 nM, and nuclease-free PCR-grade water to adjust the final volume. The *Actin* genes of *Trichoderma*, *Arabidopsis*, rapeseed, and tomato were used as internal controls for gene expression normalization, and their corresponding primer pairs are indicated in **Table 1**. Amplifications were performed in an ABI PRISM 7000 Sequence Detection System (Applied Biosystems, Foster City, CA, USA) programmed for 40 cycles under the following conditions: denaturation, 95°C for 15 s; annealing, 60°C for 1 min; extension, 72°C for 1 min. Each PCR was performed in triplicate by using the DNA extracted from the roots collected (3 sets of 5 plants for each condition per plant type). Cycle threshold values served to calculate the amount of fungal DNA using standard curves. Values of *Trichoderma* DNA were referred to the amount of *Arabidopsis*, rapeseed, or tomato DNA in every corresponding sample.

### Abiotic Stress Conditions

Wild-type and *Thkel1* rapeseed plants were watered with a 200 mM NaCl solution every 2 days, starting from the development of the third true leaf (3-week-old plants) until the end of its development cycle, in the case of salt stress or with a progressive reduction of watering in the case of drought stress.

### Biotic Stress Test

For the biotic stress test, the phytopathogenic fungal strains *B. cinera* B05.10, isolated from a grapevine field from Cádiz (Spain), and *P. lingam* CRD13/125/99, facilitated by the Regional Diagnostic Center of the Regional Government of Castile and Leon (Salamanca, Spain) and isolated from a rapeseed field from Palencia (Spain), were used. The strains were maintained in the same way as in the case of *Trichoderma* strains.

The tests of infection with the necrotrophic fungus *B. cinerea* were performed on leaves of the *A. thaliana* wild-type ecotype Col-0 and the transgenic line AtKel2 on filter paper on Petri dishes saturated with water. This was done in order to maintain the humidity in the microenvironment close to 90%, and the dishes were also sealed using parafilm. The inoculation of this pathogen was carried out by positioning a drop of 5 µl of a germination solution containing 1000 spores of *B. cinerea* (20 mM glucose, 20 mM KH_2_PO_4_, pH 6.5 adjusted by KOH). The dishes were placed in a light chamber (Fitotron AGP-1400-HR, Radiber SA, Barcelona, Spain) with a photoperiod of 16 h of light (80–100 E/m^2^/s) and 8 h of darkness at a temperature of 22°C and a relative humidity of 40%–50%. For in planta infection the methodology was similar, except that the plants are kept in transparent plastic compartments with high humidity.

The inoculation of rapeseed leaves was performed with the pathogen *P. lingam*. An agar plug of the fungus was obtained from the edge of a colony of a 7-day-old PDA culture. The plug was deposited onto detached leaves. The fifth and sixth leaves were inoculated when the plant had begun to develop the eighth leaf and then they were placed under high humidity conditions, like those of *Arabidopsis*.

In all cases, data were obtained from three biological replicates with five plants per replicate for each condition.

### Gene Expression Studies

Two leaves were collected from three sets of *Arabidopsis* plants per assayed condition, and each set included five plants. The pooled leaves were used for RNA extraction with the TRI reagent (Ambion, Austin, TX, USA), following the manufacturer’s instructions. Root RNA extraction was performed following a similar protocol with pooled samples from five plants. The cDNA synthesis was performed as previously described (Rubio et al., 2017). Gene expression was analyzed by reverse transcription PCR (RT-qPCR). PCR mixtures and amplification conditions were as previously described (Montero-Barrientos et al., 2010). The primers used are given in **Table 1**, and the *Actin* gene was used as the *Arabidopsis* endogenous control. Data are expressed using 2^−ΔΔCT^ method (Livak and Schmittgen, 2001).

### Myrosinase Activity

Total protein was extracted from 300 mg of plant material, powdered in a chilled mortar on ice, with 1 ml of 30 mM citrate-phosphate buffer, pH 7.0, containing 1 mM EDTA. The homogenate was centrifuged at 16,400 rpm for 4 min at 4°C. Supernatant was transferred to a clean tube and the protein concentration was determined (Bradford, 1976). Protein extracts from different plant samples were assayed for myrosinase activity following a sinigrin-based procedure previously described (Charron and Sams, 2004). The myrosinase activity was calculated as units (U) of activity, based on the amount of enzyme that causes the disappearance of 1 µmol of sinigrin per min, and expressed in units per gram of protein.

### Statistical Analysis

The statistical analysis of the data was carried out with the Statistix 8.0 software. Student’s t-test was used for comparison of means at *P* < 0.05; significant differences are denoted using an asterisk. One-way ANOVA using Tukey’s multiple range test at *P* < 0.05 was used for pairwise comparisons; the different letters indicate the significant differences. In the case of **Figure S1**, since no expression was detected in wild ecotypes, a log (*x* + 1) transformation was used on gene expression data so that it would meet parametric statistical assumptions.

## Results

### Defense Responses of *Thkel1*-Expressing Plants Against Biotic Stress

**Figure 1:**
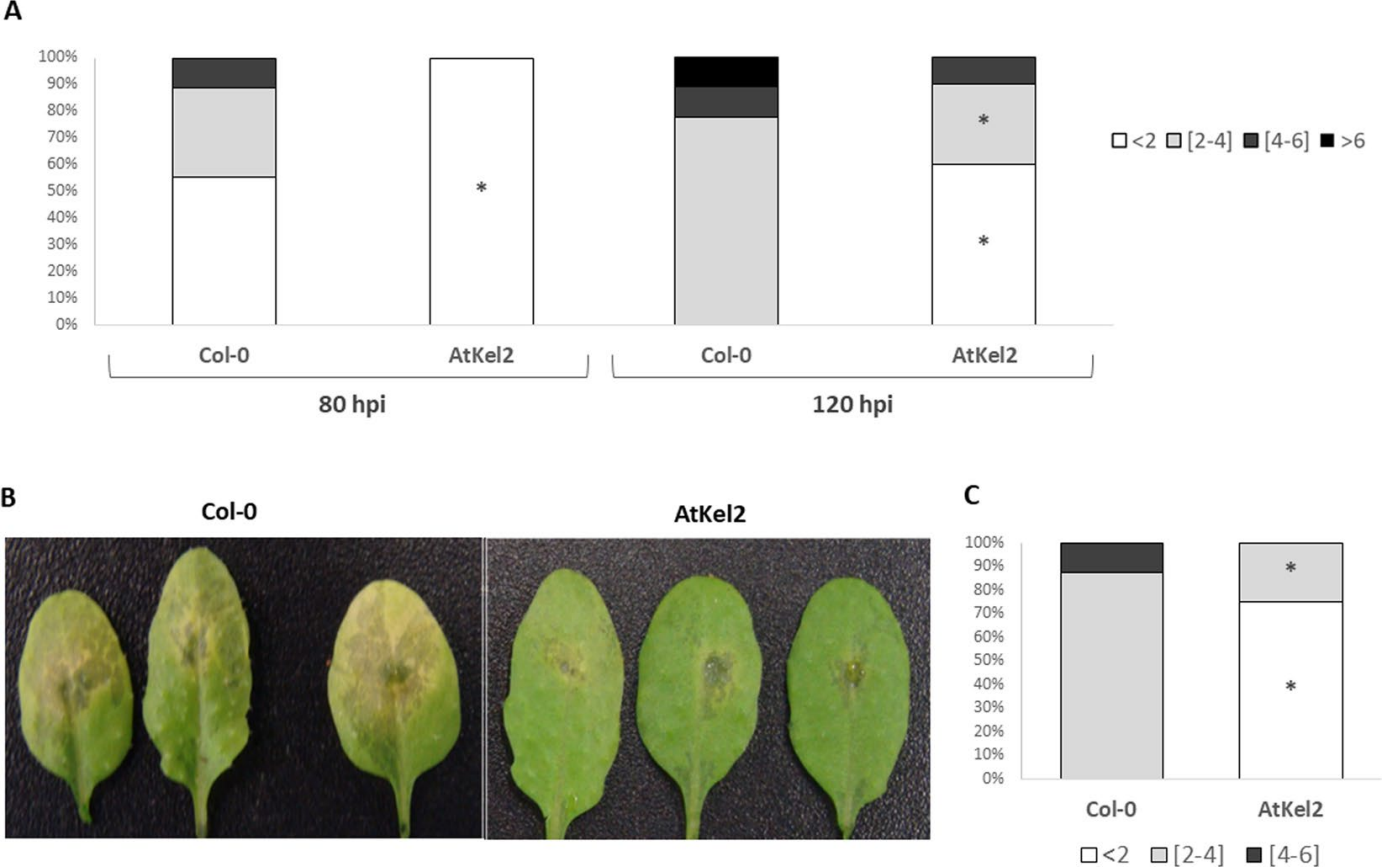
*A. thaliana* leaf lesions caused by *B. cinerea*. Leaf bioassay performed on detached leaves of Col-0 and transgenic line AtKel2 at 80 h and 120 h after *B. cinerea* inoculation **(A)**. In planta assay performed on Col-0 and transgenic line AtKel2 at 120 h after pathogen inoculation **(B** and **C)**. The quantification of the fungal lesions (A in detached leaf assay, and C in planta assay) is represented by columns that show the percentages of the diameter (mm) lesion groups. Data were obtained from three biological replicates with five plants per replicate for each condition. Asterisks denote significant differences at *P* ≤ 0.05 using the non-parametric Friedman’s test.

**Figure 2:**
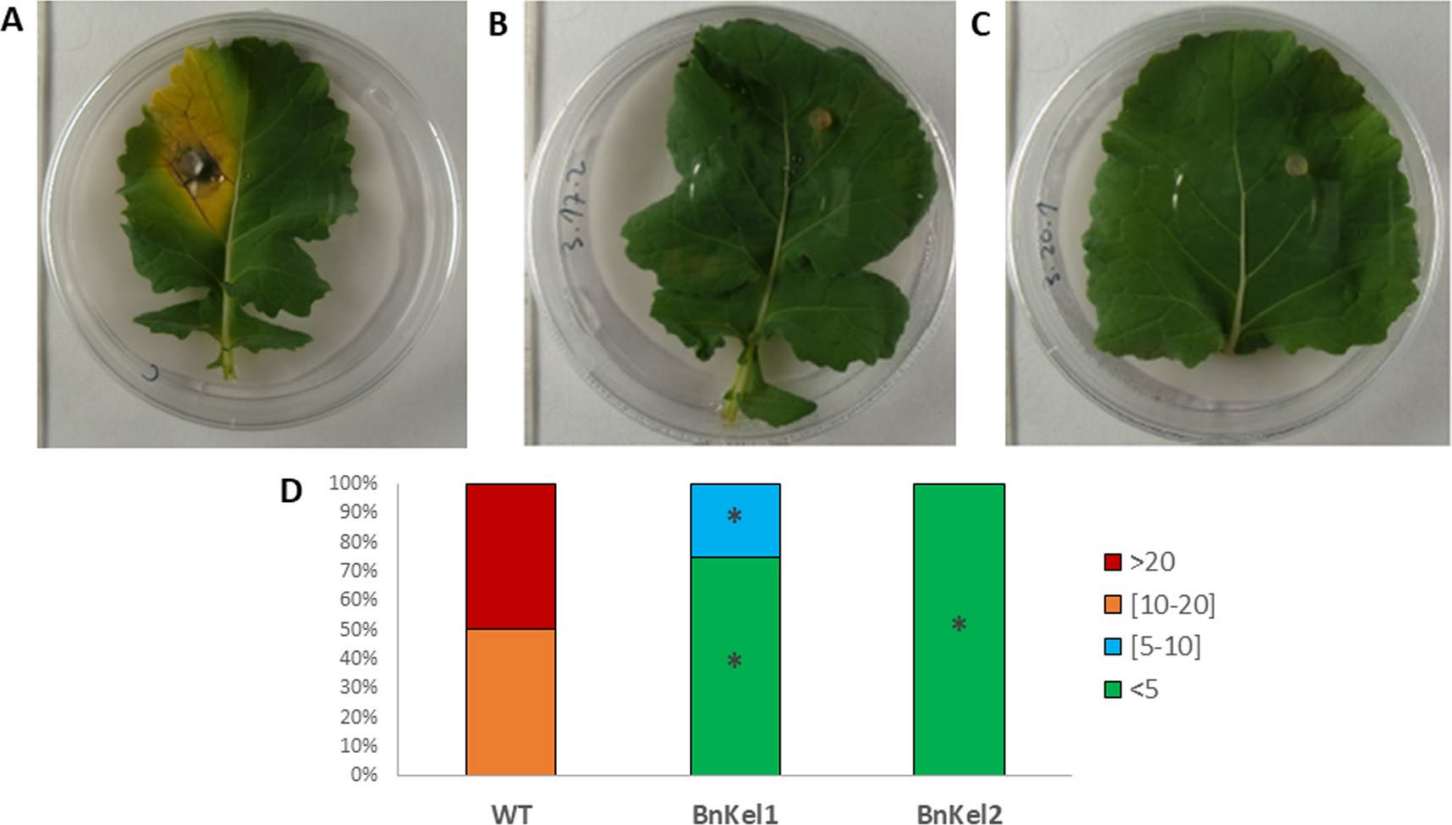
Rapeseed leaf lesions caused by *P. lingam*. Commercial variety (WT) **(A)** and transgenic lines BnKel1 and BnKel2 **(B** and **C)**, 7 days after pathogen inoculation. The quantification of the lesions produced **(D)** has been represented by columns that show the percentages of the diameter (mm) lesion groups of the lesions. Data were obtained from three biological replicates, and five plants per replicate for each condition. Asterisks denote signifiant differences at *P* ≤ 0.05 using the non-parametric Friedman’s test.

In previous work (Hermosa et al., 2011), we observed that *Arabidopsis* plants expressing the *Thkel1* gene from *T. harzianum* were more tolerant to abiotic stress conditions. To analyze plant responses to biotic stress, leaf inoculations with the foliar pathogen *B. cinerea* were performed. As shown in supplementary **Figure S2**, *B. cinerea* B05.10 produced small lesions with moderate leaf senescence in detached Col-0 *Arabidopsis* leaves. The diameter of these chlorotic regions at 120 h after inoculation with this pathogen was significantly higher compared with the leaves of AtKel2 plants expressing the *Thkel1* gene of *T. harzianum* (**Figures 1** and **S2**). Significantly larger lesions were detected in Col-0 *Arabidopsis* leaves tested in an in planta assay (**Figure 1C**). Similar results were observed in the case of rapeseed plants inoculated with the foliar pathogen *P. lingam*. A significant reduction in the size of the lesions caused by this pathogen was observed in transgenic rapeseed BnKel1 and BnKel2 plants expressing the *Thkel1* gene, compared with their wild-type counterpart (**Figure 2**).

**Figure 3:**
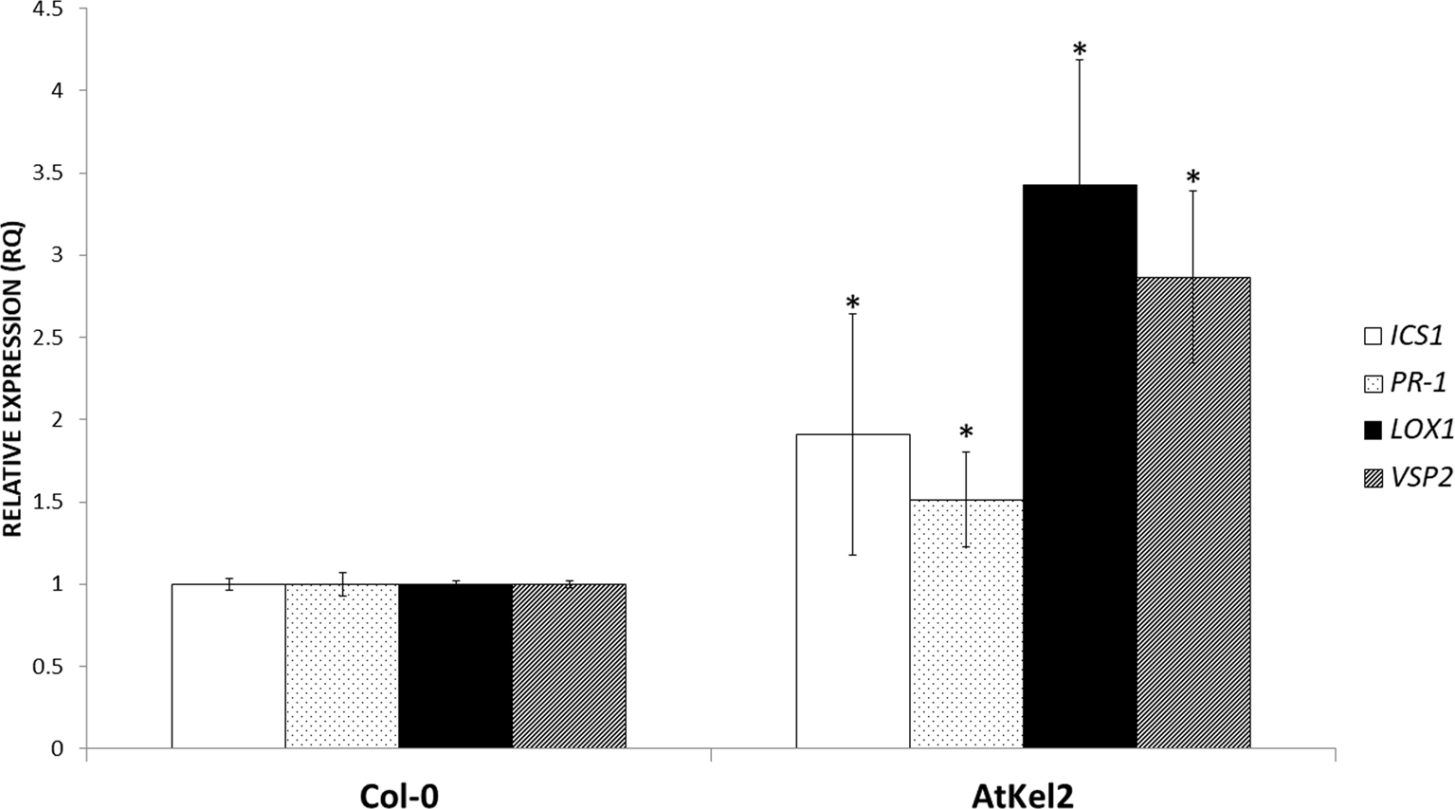
Quantitative reverse transcription polymerase chain reaction (RT-qPCR) analysis of the expression of some defense genes in the leaves of *A. thaliana* wild-type plants (Col-0) and the transgenic line AtKel2 (5-week-old plants). Genes of the isochorismate synthase 1 (*ICS1*), pathogenesis-related protein 1 (*PR-1*), lipoxygenase 1 (*LOX1*), and vegetative storage protein (*VSP2*). Values correspond to relative measurements against Col-0 (2^–ΔΔCt^ = 1). The *Arabidopsis actin* gene was used as an internal reference gene. Data are the mean of three biological replicates for each condition with the corresponding standard deviation, and for each biological replicate and condition, two leaves per plant from five plants were used. Student’s t-test was performed. Asterisks denote significant differences at *P* ≤ 0.05.

**Figure 4:**
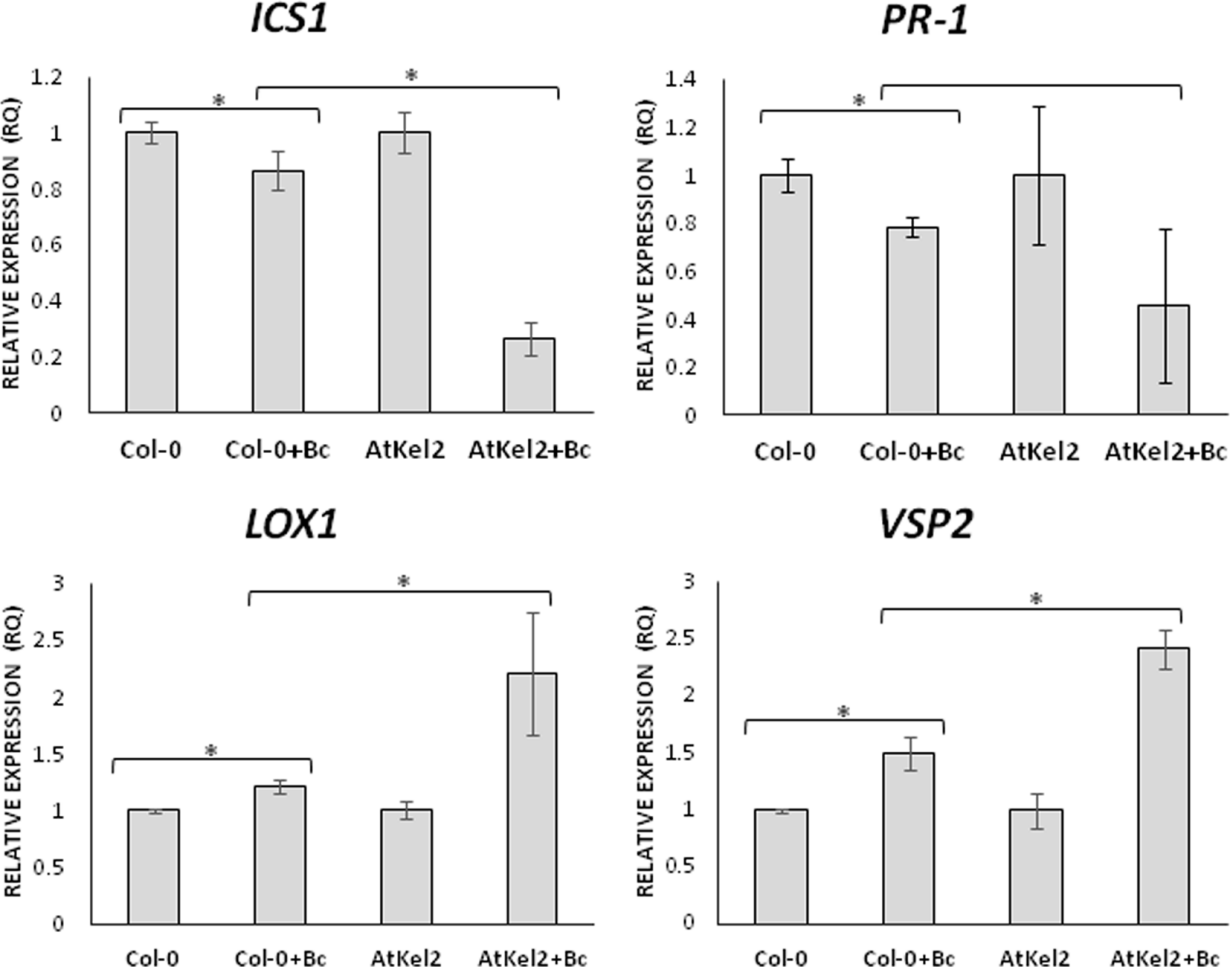
Quantitative reverse transcription polymerase chain reaction (RT-qPCR) analysis of the expression of some defense genes in the leaves of *A. thaliana* wild type (Col-0) and the transgenic line AtKel2 infected with *B. cinerea*, 120 h after infection (+Bc). Genes of the isochorismate synthase 1 (*ICS1*), pathogenesis-related protein 1 (*PR-1*), lipoxygenase 1 (*LOX1*) and vegetative storage protein (*VSP2*). Values correspond to relative measurements against plants without infection (2^–ΔΔCt^ = 1). The *Arabidopsis actin* gene was used as an internal reference gene. Data are the mean of three biological replicates for each condition with the corresponding standard deviation, and for each biological replicate and condition, three leaves per plant from five plants were used. Student’s t-test was performed. Asterisks denote significant differences at *P* ≤ 0.05.

In view of these results, we analyzed the expression of defense-related genes implicated in both the biosynthesis and responses dependent on the phytohormones salicylic acid (SA) and jasmonic acid (JA); *ICS1* (SA biosynthesis); *PR-1* (SA-dependent defense); *LOX1* (JA biosynthesis); and *VSP2* (JA-dependent defense). A significant increase in the expression levels of the four genes analyzed was observed in the leaves of 5-week-old AtKel2 *Arabidopsis* plants compared with the wild type Col-0 (**Figure 3**). In addition, we also analyzed by RT-qPCR the profile of these defense markers genes in *Arabidopsis* plants challenged with the necrotrophic pathogen *B. cinerea* 120 h after infection. In this case, a significant increase in the expression levels of JA-related genes was observed in AtKel2 plants, whereas a reduction in the expression of SA-related genes was detected (**Figure 4**).

#### The Role of the *Thkel1* Gene in Root Colonization

**Figure 5:**
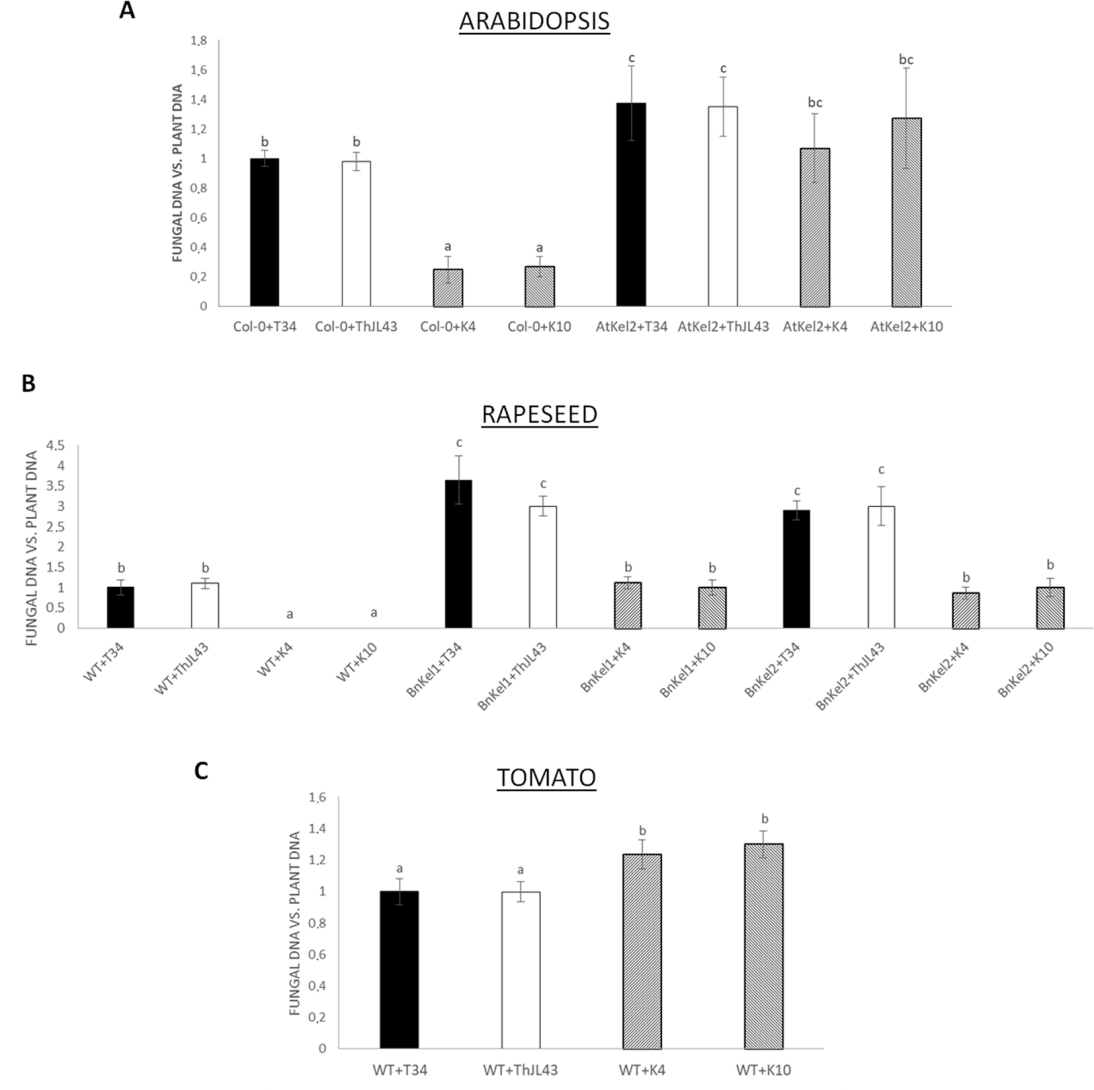
Analysis of root colonization by *T. harzianum* (T34: control strain; ThJL43: transformation control; K4 and K10: silenced transformats for the *Thkel1* gene) in hydroponic culture of *A*. *thaliana* **(A)**, rapeseed **(B)**, and tomato **(C)** in a greenhouse. To quantify *Arabidopsis*, rapeseed, and tomato root colonization, the DNA of the fungus was quantified by qPCR from radicular samples using the *actin* genes from both the plants and the fungus. Data are the mean of three biological replicates for each condition with the corresponding standard deviation, and for each biological replicate and condition, roots from five plants were used. One-way analysis of variance (ANOVA) was performed, followed by the Tukey’s test. Different letters represent significant differences (*P* < 0.05).

In order to characterize the role of *Thkel1* of *T. harzianum* T34 in the colonization of Brassicaceae and non-Brassicaceae plants, the fungal levels of the wild-type strain (T34) and the silenced *Thkel1* mutants (K4 and K10) strains were determined in *Arabidopsis*, rapeseed, and tomato plants, as well as in *Arabidopsis* (AtKel2) and rapeseed (BnKel1 and BnKel2) *Thkel1* transgenic plants. The latter were generated in this particular study. To this end, we conducted a root colonization study in hydroponic culture (Alonso-Ramírez et al., 2014) using previously obtained *Thkel1* silenced transformants of *T. harzianum* (Hermosa et al., 2011). No differences were detected in root colonization levels when the *Trichoderma* wild-type T34 strain and the transformation control ThJL43 strain were used. Interestingly, a significant reduction in the degree of root colonization was detected when *Arabidopsis* Col-0 plants were inoculated with the *Trichoderma* K4 and K10 silenced transformants. Furthermore, colonization was restored when K4 and K10 interacted with AtKel2 plants overexpressing *Thkel1*, although it was not completely restored when compared to *Arabidopsis* wild-type (**Figures 5A** and **S3**). A similar root colonization profile was observed in rapeseed plants (**Figure 5B**), although in this case no colonization at all was detected when wild-type plants were inoculated with the silenced transformants. By contrast, the highest degree of tomato root colonization was detected in the interaction with the *Thkel1* silenced transformants (**Figure 5C**).

**Figure 6:**
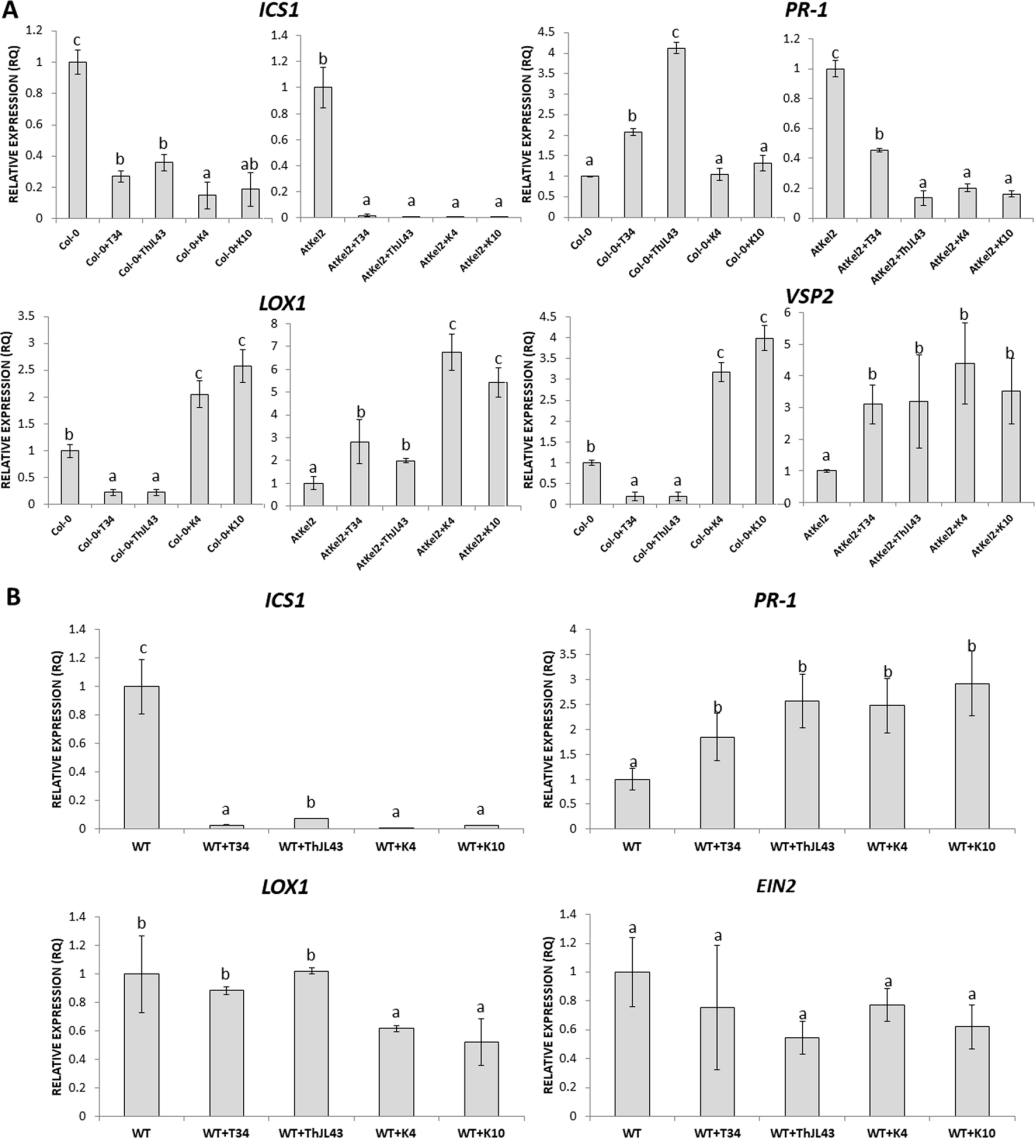
Quantitative reverse transcription polymerase chain reaction (RT-qPCR) analysis of the expression of some defense genes in the roots of *A. thaliana* wild type (Col-0) and transgenic line (AtKel2) plants **(A)** and in tomato WT roots **(B)** inoculated with the *T. harzianum* strains T34 (control), ThJL43 (transformation control), K4 or K10 (*Thkel1* gene silenced transformants). Genes of the isochorismate synthase 1 (*ICS1*), pathogenesis-related protein 1 (*PR-1*), lipoxygenase 1 (*LOX1*), vegetative storage protein (*VSP2*), and ethylene signaling protein (*EIN2*). Values correspond to relative measurements against plants without infection (2^–ΔΔCt^ = 1). The *Arabidopsis actin* gene and the tomato actin *gene* were used as an internal reference gene. Data are the mean of three biological replicates for each condition with the corresponding standard deviation, and for each biological replicate and condition, roots from five plants were used. One-way analysis of variance (ANOVA) was performed, followed by the Tukey’s test. Different letters represent significant differences (*P* < 0.05) between plants with and without fungal inoculation.

Additionally, the expression of defense marker genes was analyzed in *Arabidopsis* roots of both wild-type and transgenic plants (**Figure 6A**). Although *ICS1* expression levels were significantly reduced in the wild-type plants interacting with the K4 and K10 silenced transformants, compared with those observed in the interaction with T34, it is noteworthy that the expression of the SA-dependent defense *PR-1* gene was significantly increased in the interactions between Col-0 plants and strain T34 and the expression was even greater between Col-0 and strain ThJL43. However, reduced expression levels of *PR-1* gene were detected in the transgenic line AtKel2 when challenged with K4 and K10 in comparison with those challenged with T34 or ThJL43 strains. Regarding JA-related genes, a significant decrease in their expression levels was observed during the interactions between the wild-type plants and T34, or the wild type and ThJL43. On the contrary, when *Arabidopsis* wild-type plants were challenged with the silenced transformants K4 or K10, the expression levels of JA-related genes were significantly increased. In the case of the interaction of *Thkel1*-overexpressing plants with *Trichoderma*, a significant increase in the expression levels of the JA-related genes was detected, with the highest levels being detected in AtKel2 plants challenged by the *T. harzianum* silenced mutants K4 and K10. In the case of tomato plants (**Figure 6B**), the expression levels of *ICS1* were significantly reduced in all cases in comparison with those observed in wild-type plants whereas a significant increase in *PR-1* levels was detected in tomato plants challenged with any of the *Trichoderma* strains. Moreover, a significant reduction in *LOX1* levels was observed in tomato plants inoculated with the silenced transformants while the expression of *EIN2*, a major regulator of the ET signaling pathway, did not significantly change.

#### Systemic Responses in Leaves of *Arabidopsis* Plants Inoculated With *T. harzianum*

**Figure 7:**
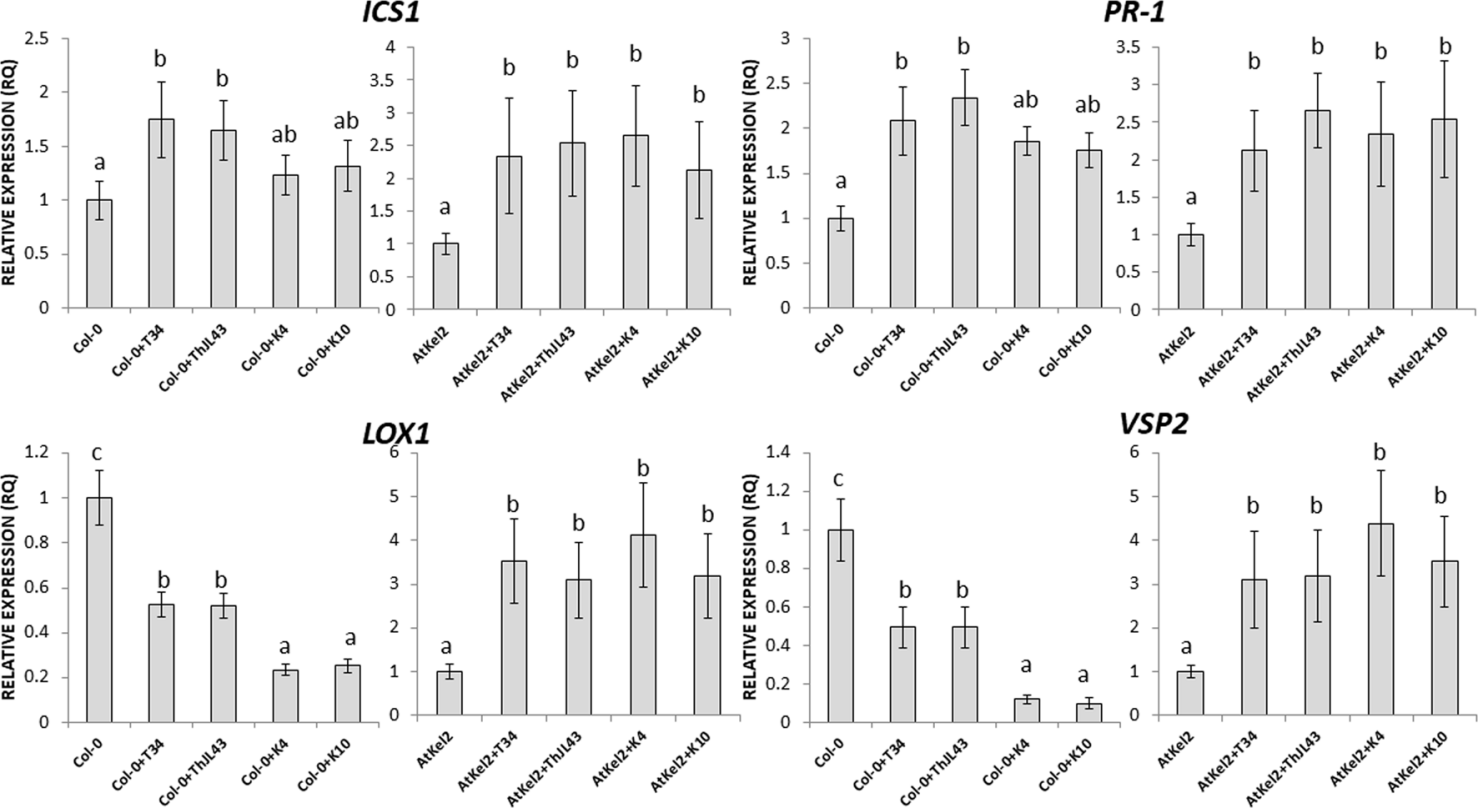
Quantitative reverse transcription polymerase chain reaction (RT-qPCR) analysis of the expression of some defense genes in the leaves of *A. thaliana* wild type (Col-0) and transgenic line (AtKel2) plants, after inoculation with the control strain (+T34), the transformation control (+ThJL43), and the transformants with the silenced *Thkel1* gene (+K4 and +K10) of *T. harzianum*. Genes of the isochorismate synthase 1 (*ICS1*), pathogenesis-related protein 1 (*PR-1*), lipoxygenase 1 (*LOX1*), vegetative storage protein (*VSP2*), and ethylene signaling protein (*EIN2*). Values correspond to relative measurements against plants without inoculation (2^–ΔΔCt^ = 1). The *Arabidopsis actin* gene was used as an internal reference gene. Data are the mean of three biological replicates for each condition with the corresponding standard deviation, and for each biological replicate and condition, two leaves from three plants were used. One-way analysis of variance (ANOVA) was performed, followed by the Tukey’s test. Different letters represent significant differences (*P* < 0.05) between plants with and without fungal inoculation.

In order to assess the systemic responses in leaves of Col-0 and AtKel2 plants challenged with the different *Trichoderma* strains (**Figure 7**), we calculated the expression levels of the same defense-marked genes used so far. A significant *ICS1* expression increase was observed in Col-0 plants inoculated with T34 and ThJL43, whereas no changes were detected in these plants challenged with the silenced transformants. In addition, a significant increase in *ICS1* expression was detected in Atkel2 leaves when these *Thkel1*-overexpressing plants were inoculated with any of the *Trichoderma* strains. Similar results were observed in the case of *PR-1*. Concerning JA-markers, a significant *LOX1* and *VSP2* expression decrease was observed in Col-0 leaves after *Trichoderma* inoculation. This decline was more dramatic in the case of K4 and K10 silenced transformants. In contrast, a significant increase in the expression levels of these two genes was detected in leaves of Atkel2 plants.

#### Myrosinase Activity

**Figure 8:**
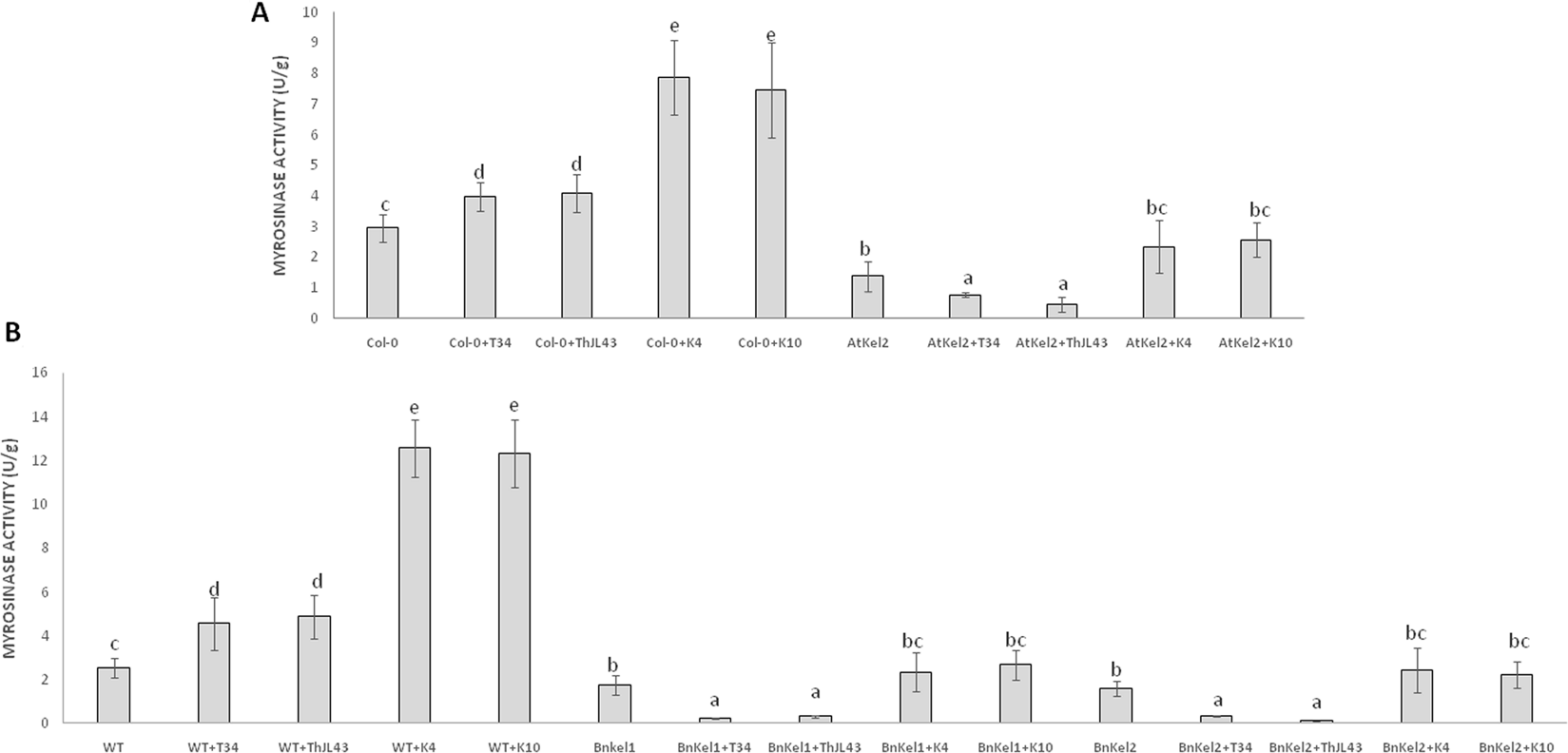
Myrosinase activity (U/g of protein) in roots of *A. thaliana* wild type (Col-0) and transgenic line (AtKel2) plants **(A)** and rapeseed commercial variety (WT) and transgenic lines (BnKel1 and BnKel2) plants **(B)**, after inoculation with the control strain (+T34), the transformation control (+ThJL43), and the transformants with the silenced *Thkel1* gene (+K4 and +K10) of *T. harzianum*. Data are the mean of three biological replicates for each condition with the corresponding standard deviation, and for each biological replicate and condition, roots from five plants were used. One-way analysis of variance (ANOVA) was performed, followed by the Tukey’s test. Different letters represent significant differences (*P* < 0.05) between plants with and without fungal inoculation.

*Thkel1* shares sequence similarity to NSPs and ESPs that modulate myrosinase activity in Brassicaceae plants. Thus, we analyzed this enzymatic activity in *Arabidopsis* and rapeseed plants challenged with the different *Trichoderma* strains (**Figure 8**). A significant myrosinase activity increase was observed in *Arabidopsis* plants inoculated with *Trichoderma*. The augmentation was significantly greater in the case of the K4 and K10 silenced transformants. In contrast, a significant decrease in this activity was detected in Atkel2 in comparison with Col-0 plants. This reduction was more dramatic in Atkel2 plants challenged with the strains T34 and ThJL43. Similar results were observed in the case of wild type, Bnkel1 and Bnkel2 rapeseed plants.

#### Productivity

**Figure 9:**
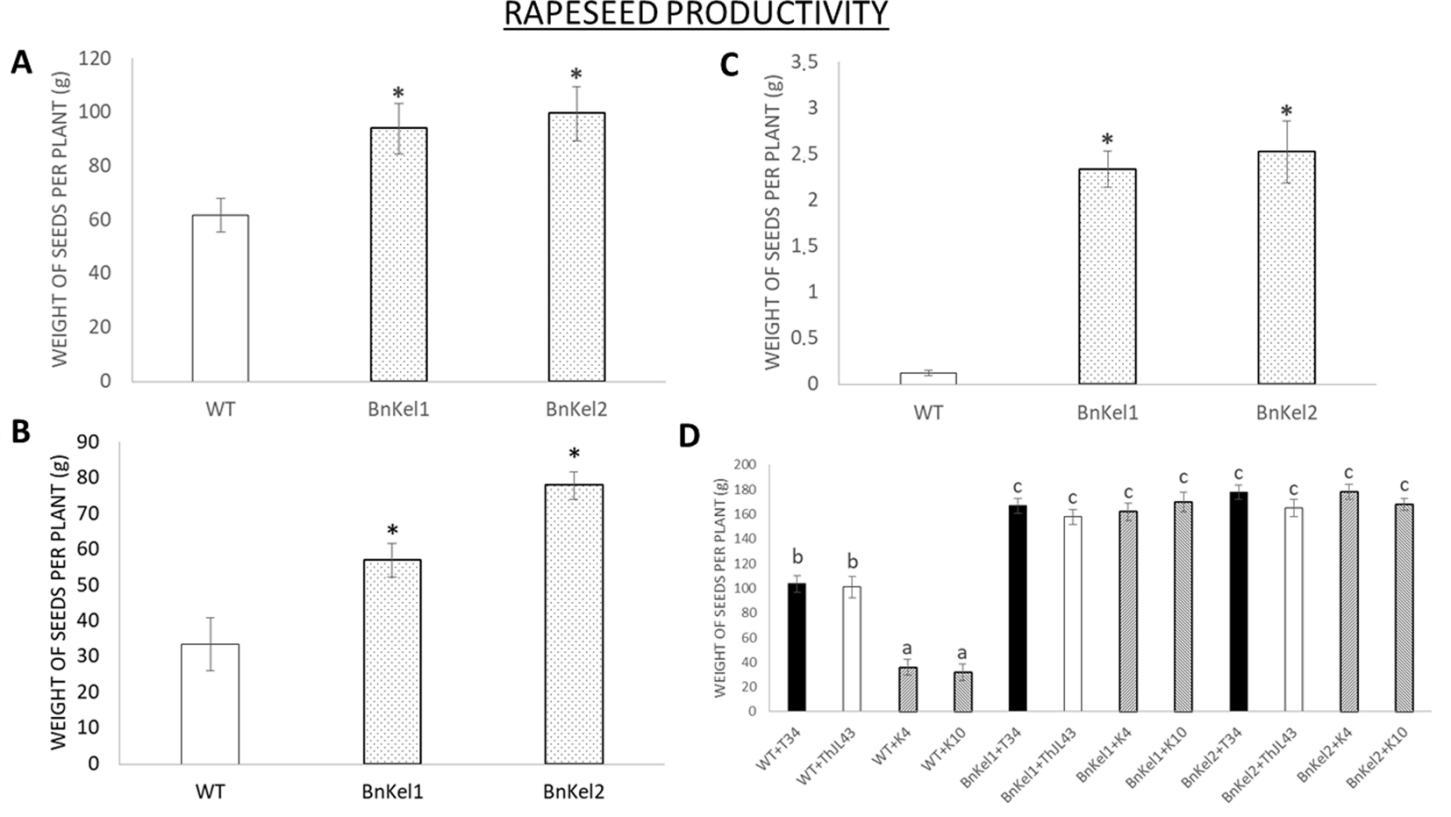
Rapeseed productivity. The weight of the seeds collected from the commercial variety (WT) and the transgenic *Thkel1* lines (BnKel1 and BnKel2) per plant without stress **(A)**, under salt stress (NaCl 200 mM; **B**), and under drought stress **(C)**. The weight of seeds collected after inoculation with the control strain (+T34), the transformation control (+ThJL43), and the transformants with the silenced *Thkel1* gene (+K4 and +K10) of *T. harzianum* **(D)**. Data are the mean of three biological replicates for each condition with the corresponding standard deviation, and 15 plants per replicate were used. Student’s t-test was performed; asterisks denote significant differences at *P* ≤ 0.05. One-way analysis of variance (ANOVA) was performed, followed by the Tukey’s test in *T. harzianum* inoculation. Different letters represent significant differences (*P* < 0.05).

Rapeseed is one of the most important oilseed crops. Thus, we analyzed productivity in both the wild-type and *Thkel1*-expressing rapeseed plants. The seed weight per plant was significantly higher in the transgenic lines BnKel1 and BnKel2 than that measured for the wild-type plants (**Figure 9A**) and was also the case under salt (**Figure 9B**) and drought stress conditions (**Figure 9C**). Moreover, the higher degree of *T. harzianum* colonization of BnKel1 and BnKel2 rapeseed roots was accompanied by an increase in productivity (**Figure 9D**). The wild-type rapeseed plants, on the other hand, challenged with the *Thkel1* silenced transformants K4 and K10 showed significantly lower silique production and seed weight values compared to those obtained for wild-type plants colonized with the *T. harzianum* control strains, the wild type T34, and the transformation control ThJL43.

## Discussion

Plant transformation with *Trichoderma* genes has been shown to improve plant responses to both biotic and abiotic stress conditions (Nicolás et al., 2014). In previous work, we reported that the expression of the *Thkel1* from *T. harzianum* enhanced *Arabidopsis* plant responses to salt and osmotic stresses (Hermosa et al., 2011) through an increase in β-glucosidase activity. The *Thkel1* gene codes for a protein with five Kelch domains. This class of proteins has been involved in several plant processes including circadian clock, flowering, hypocotyl elongation, and plant defense (Hassan et al., 2015).

In the present work we have generated rapeseed transgenic lines able to express the *Thkel1* gene (**Figure S1**), in addition to the already available *Arabidopsis* AtKel2 plants (Hermosa et al., 2011), and analyzed the responses of these plants to biotic stress. The resulting transgenic rapeseed BnKel1 and BnKel2 lines as well as the AtKel2 line were more resistant than the wild type to the foliar pathogens *P. lingam* and *B. cinerea* (**Figures 1**, **2**, and **S2**). Due to the aggressiveness of B05.10 strain of *B. cinerea*, one would expect more severe lesions in wild type plants. However, it has been reported that different lesion traits were detected in *Arabidopsis* leaves depending on the *B. cinerea* strain used (Fordyce et al., 2018), including B05.10. In any case, the symptoms and the chlorotic regions observed in detached leaves of wild-type *Arabidopsis* plants were not observed in AtKel2 plants.

To investigate the cause of this increased resistance to foliar pathogens in the different *Thkel1* overexpressing lines, we analyzed the expression of several defense marker genes in *Arabidopsis*. The expression levels of SA- and JA- biosynthesis and defense related genes were significantly increased in the AtKel2 transgenic line compared to those recorded for Col-0 plants (**Figure 3**). Moreover, a significant increase in JA-related genes was detected when *Arabidopsis* plants were challenged with *B. cinerea*, whereas the SA-related gene expression was significantly reduced (**Figure 4**). These results are consistent with the size of the chlorotic lesion caused by *B. cinerea* in Col-0 compared with that observed in AtKel2 plants infected with this pathogen. The putative role of proteins with Kelch domains in plant defense responses has been widely studied (Kim and Delaney, 2002; Guo and Stotz, 2007; Thiel et al., 2012). In addition, β-glucosidase activity is also involved in plant defense responses (Opassiri et al., 2010) and it has been reported that proteins with Kelch domains are able to decrease SA responses (Zhang et al., 2013; Zhang et al., 2015). These findings are in line with what was observed in AtKel2 plants artificially inoculated with plant pathogens, such as *Botrytis* and *Phoma*, as well as the increase in the antagonistic JA response (**Figure 4**).

The next step was to evaluate the degree of root colonization by *T. harzianum* in *Thkel1*-expressing and non-expressing plants and to assign a role for this gene in this process (**Figures 5** and **S3**). In both *Arabidopsis* and rapeseed *Thkel1*-expressing plants, a significant increase in root colonization by T34 was detected. Conversely, when the corresponding *Arabidopsis* and rapeseed wild-type plants were challenged by the silenced transformants K4 and K10, a dramatic reduction in root colonization was observed. This effect was partially restored in the interaction between these silenced transformants and the transgenic AtKel2, BnKel1, or BnKel2 plants (**Figures 5A, B**). These findings suggest *Thkel1* plays a key role in root colonization in Brassicaceae, which is supported by the fact that the results recorded for the tomato plants were completely different (**Figure 5C**). In this case, the greatest degree of root colonization was observed for *ThKel1*-silenced mutants. Although we cannot currently explain this result, it indicates that this gene is not likely to be involved in *Trichoderma* root colonization in non-Brassicaceae species.

Several *Trichoderma* genes have been shown to play important roles in plant root colonization. For instance, the swollenin *TaSwo* gene from *T. asperellum* remarkably increases the ability of overexpressing transformants to colonize cucumber roots, whereas its silencing reduces this ability (Brotman et al., 2008). In addition, two aspartyl proteases and an hydrophobin from *T. asperellum* were also identified as pivotal genes in the root colonization process (Viterbo et al., 2004; Viterbo and Chet, 2006). The endopolygalacturonase ThPG1 from *T. harzianum* was reported as a key player in the root colonization while activating SA-dependent defense responses in tomato (Morán-Diez et al., 2009) and *Thkel1* could be another *Trichoderma* gene responsible for root colonization. However, in this case *Thkel1* proved itself useful in the colonization of the Brassicaceae root system.

The differences in root colonization observed among *T. harzianum* strains are consistent with the expression profiles of defense-related genes detected in the roots of Col-0, AtKel2, and tomato plants (**Figure 6**). As expected, since SA is the key phytohormone regulating the *Trichoderma* root colonization process (Alonso-Ramírez et al., 2014), a significant increase in *PR-1* levels was observed in Col-0 challenged by the *T. harzianum* wild-type strain, as well as in the case of the transformation control strain. This increase in *PR-1* levels was however not detected in the case of K4 and K10 strains. Since these two silenced transformants were not successful enough in colonizing Col-0 roots, the activation of SA pathway would not appear to be required. This increase was also not detected in the AtKel2 plants. In this specific case, a significant increase in the expression of JA-marker genes, such as *LOX1* and *VSP2*, was detected, as well as a reduction in the expression of SA-related genes. These results highlight the involvement of a Kelch domain protein in the JA-mediated defense response due to its ability to inhibit the SA-responsive defense through proteolytic degradation by ubiquitination pathways of SA-related proteins in *Arabidopsis* (Zhang et al., 2013; Zhang et al., 2015). Depending on the presence of the *ThKel1* gene in *Trichoderma* we have observed different degrees of root colonization between Brassicaceae and tomato plants (**Figure 5**). This result is consistent with the differences in the expression levels of the defense-marker genes analyzed in this study (**Figure 6**), and highlights, again, the role of *Thkel1* gene in the *Trichoderma* root colonization process in Brassicaceae, probably through the modulation of myrosinase activity (**Figure 8**).

Concerning systemic responses in *Arabidopsis*, the silencing of *Thkel1* gene leads to a downregulation of JA-related genes after *Trichoderma* inoculation (**Figure 7**). In view of this result, it would be expected that this mutant was not able to trigger ISR responses mediated by JA against *Botrytis* compared to those induced by *Trichoderma* wild-type strain. To confirm this observation, further research is needed. In addition, overexpression of this gene in *Arabidopsis* plants leads to an upregulation of JA as well as SA-marker genes, although in this case this upregulation was also observed in wild type plants. These results indicate induction of systemic responses, that has also been reported against *Sclerotinia sclerotiorum* in *B. napus* after root colonization by *T. harzianum* (Alkooranee et al., 2017). These authors proved that this induction of systemic defense through JA/ET and SA-signaling pathways occurs at different times, that is in agreement with our results. JA seems to be very important in *Thkel1* overexpressing plants against *B. cinerea* (**Figure 4**). By contrast, only *PR-1* is induced in *Arabidopsis* Col-0 roots upon *Trichoderma* inoculation (**Figure 6A**), an expected result, since we have previously reported the important role of SA in the root colonization process avoiding a massive fungal invasion by *Trichoderma* (Alonso-Ramírez et al., 2014). It is important to note that *PR-1* expression was higher in Col-0 roots colonized by ThJL43 as compared to those colonized by T34. This could be explained by a greater local defense activation by ThJL43 since no differences in root colonization levels were detected between both strains.

The Brassicaceae family is characterized by the presence of glucosinolates, a class of allelopathic compounds involved in plant defense (Cartea et al., 2011). The differences in root colonization between the Brassicaceae and non-Brassicaceae plants observed in this study may be associated with the production of these compounds. ThKEL1 shares a high degree of homology with NSPs and ESPs related with the metabolism of glucosinolates. Glucosinolates and myrosinases, enzymes responsible of hydrolysis, are spatially separated. Only after tissue damage is provoked by a pathogen, does glucosinolate breakdown begin. Other proteins, such as NSPs and ESPs, can modify myrosinase activity leading to the formation of other chemical compounds less toxic than isothiocyanate glucosinolates, such as nitriles or epithionitriles (Agerbirk and Olsen, 2012; Martínez-Ballesta and Carvajal, 2015). Our hypothesis is related to the possible role of *Thkel1* in the degradation of glucosinolates, which would in turn help *Trichoderma* species to colonize the Brassicaceae roots. Thus, we analyzed myrosinase activity in *Arabidopsis, B. napus* wild-type, AtKel2, and BnKel plants challenged with the different *Trichoderma* strains (**Figure 8**). In summary, an increased myrosinase activity was recorded when plants were challenged with the silenced transformants and a decrease in transgenic plants that overexpress *Thkel1* gene. These data are consistent with the degree of root colonization of the different *Trichoderma* strains (**Figure 5**). Myrosinases and glucosinolates are localized in specialized and different cells. Herbivore attack breaks these cells beginning the plant defense response (Shirakawa et al., 2016). However, it has been recently described that some atypical myrosinases, with antifungal capacities such as PEN2 or PYK10, are not accumulated in those specialized cells. These atypical myrosinases are secreted through ABC transporters and exhibit their effect at the extracellular level (Shirakawa and Hara-Nishimura, 2018). Our data suggest that the *Thkel1* gene may modulate myrosinase activity, and we can speculate that the interaction with some of these atypical myrosinase may decrease toxic glucosinolate levels, allowing root colonization by *Trichoderma* in Brassicaceae. In any case, further progress is needed in this direction to confirm this possibility.

It has been reported that some *Trichoderma* strains, such as *T. asperellum* (Kowalska, 2014) and *T. koningii* (Wang et al., 2009), were able to increase the crop yield of crucifers. Since rapeseed is one of the most economically important oilseed crops, and due to the observed differences in the degree of root colonization, we analyzed both silique and seed production in these plants. The higher degree of root colonization by *T. harzianum* in BnKel1 and BnKel2 plants was also accompanied by an increase in the number of siliques per plant and in the total seed weight per plant (**Figure 9**). Furthermore, this effect was enhanced under abiotic stress conditions, which could be expected considering the higher tolerance to salt and osmotic stresses previously reported in AtKel plants (Hermosa et al., 2011). The increase in seed yields may be related to the ability of those proteins with Kelch-repeat domains to improve the number and size of seeds, as previously described in rice (Chen et al., 2013), to the ratio of β-glucosidases in the nutritional cycle (Caruso, 2010), or to the inorganic phosphate accumulation in plants as reported in *Arabidopsis* (Malboobi and Lefebvre, 1997).

In conclusion, the results presented here show that the overexpression of *Thkel1* gene from *T. harzianum* improves plant responses to pathogens by inducing systemic defenses mediated by JA and that the *ThKel1* gene of *Trichoderma* plays a key role in colonizing the roots of Brassicaceae plants, probably through modulation of myrosinase activity. In addition, its expression in Brassicaceae increases *T. harzianum* root colonization, which is accompanied by higher plant productivity, both under control conditions and under salt or drought stress.

## Data Availability Statement

All datasets generated for this study are included in the article/**Supplementary Material**.

## Author Contributions

JP performed the experiments. JP, EM, and CN conceived and designed the experiments. JP, RH, EM, and CN analyzed the data. JP, RH, EM, and CN wrote the paper.

## Funding

This work was supported by the grants AGL2015-70671-C2-1-R from the Spanish Ministry of Economy and Competitiveness and SA230U13 and SA270P18 from the Regional Government of Castile and Leon (Spain).
