# Supplementary figures and images for: The Trichoderma harzianum Kelch Protein ThKEL1 Plays a Key Role in Root Colonization and the Induction of Systemic Defense in Brassicaceae Plants
Source: Front Plant Sci. 2019 Nov 15;10:1478. doi: 10.3389/fpls.2019.01478 (PMC6873215; doi:10.3389/fpls.2019.01478)

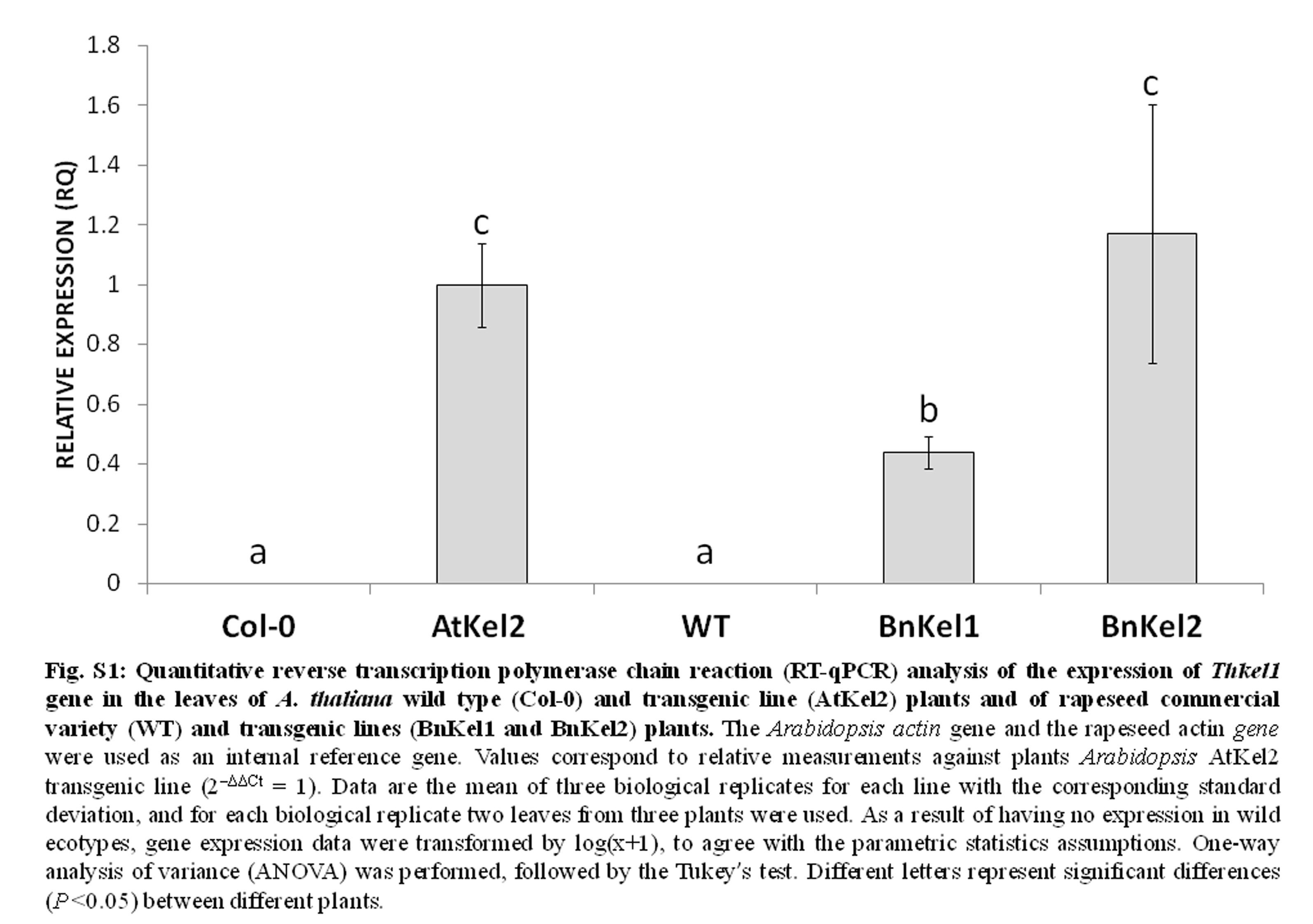

Supplement: Supplementary file 1 [file Image_1.tif]

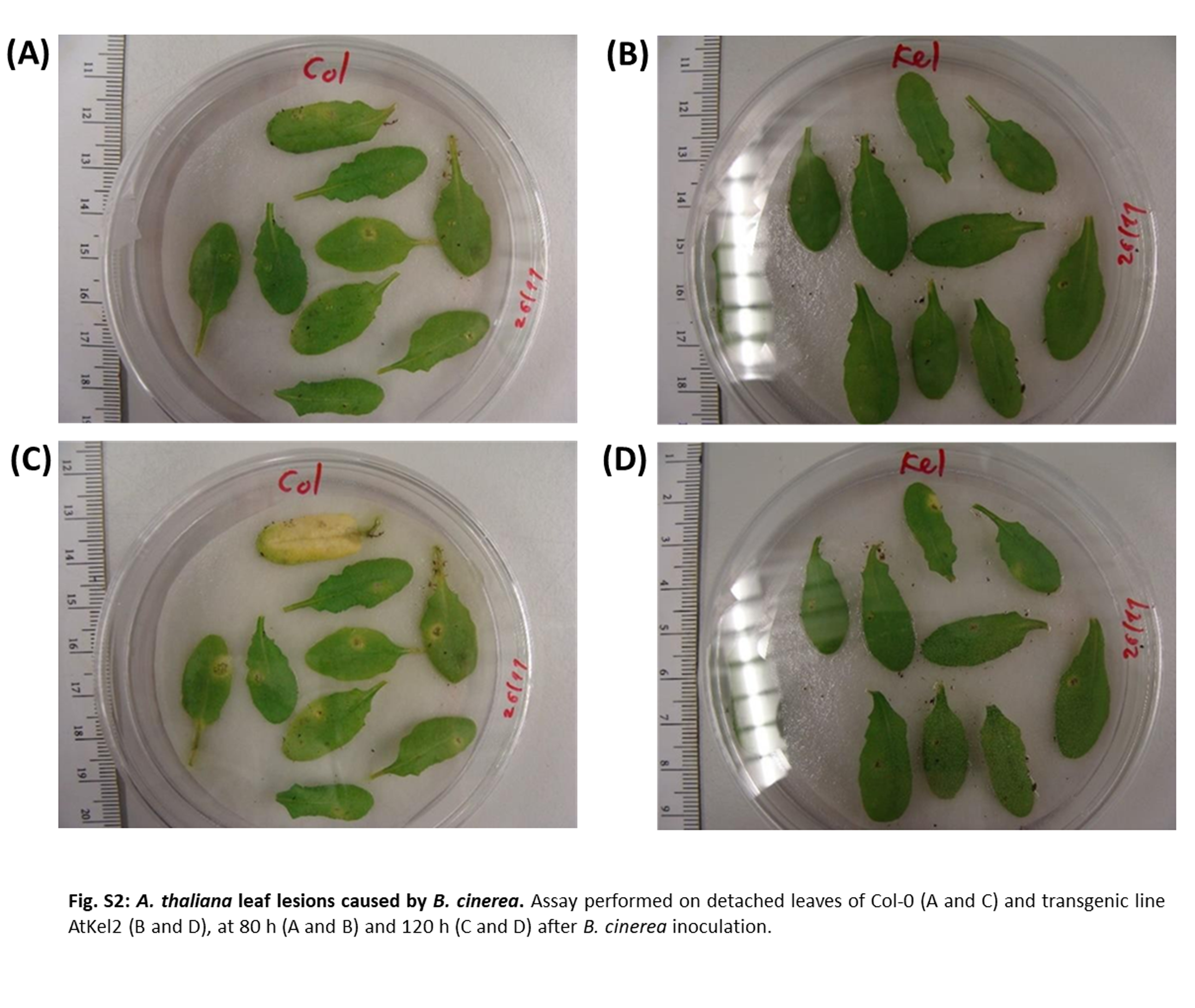

Supplement: Supplementary file 2 [file Image_2.tif]

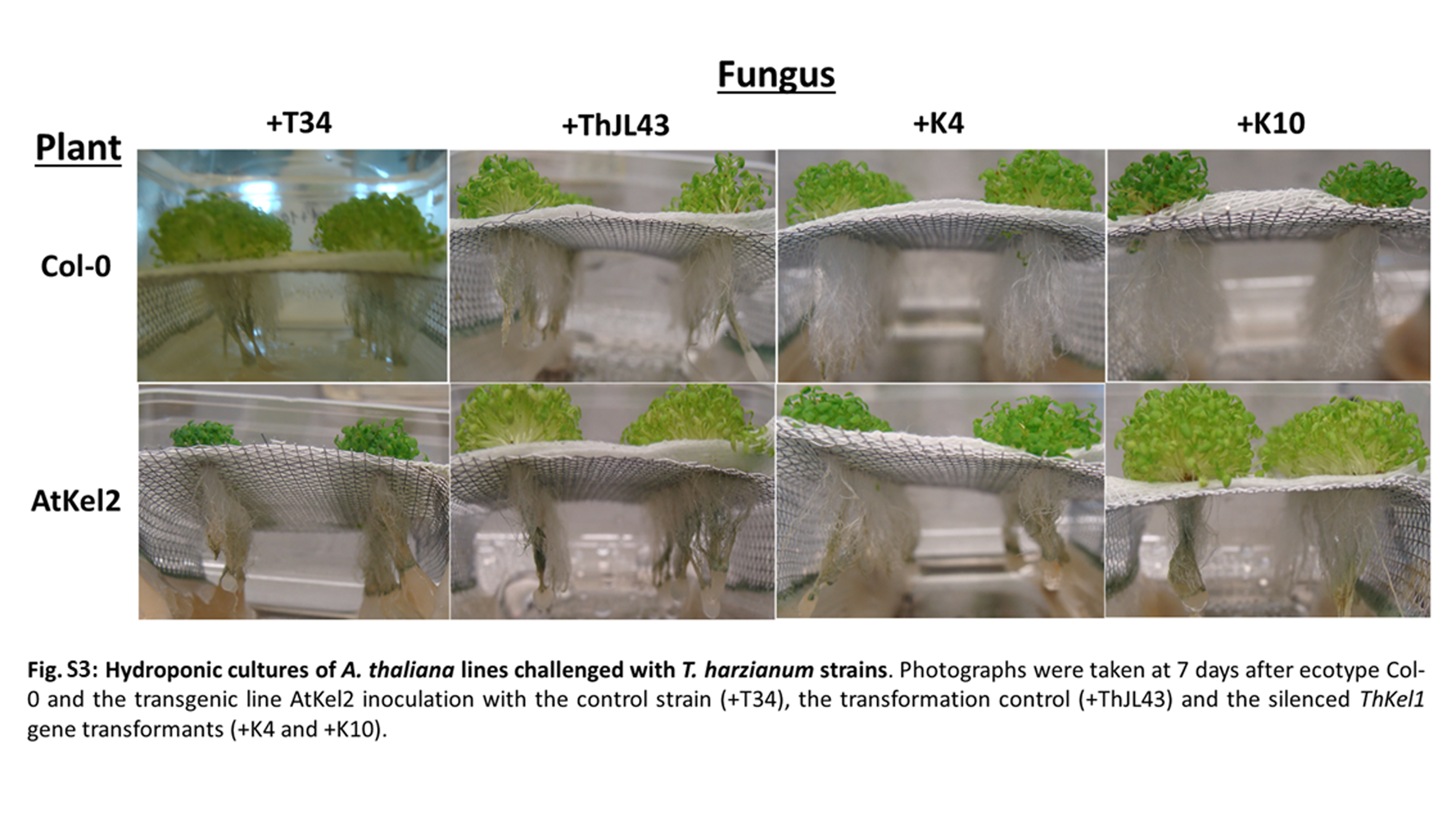

Supplement: Supplementary file 3 [file Image_3.tif]
